# The Role of the Exonic lncRNA PRKDC-210 in Transcription Regulation

**DOI:** 10.3390/ijms232213783

**Published:** 2022-11-09

**Authors:** Junling Mo, Guangyao Fan, Toshifumi Tsukahara, Matomo Sakari

**Affiliations:** Area of Bioscience and Biotechnology, Japan Advanced Institute of Science and Technology, 1-1 Asahidai, Nomi 923-1292, Ishikawa, Japan

**Keywords:** cancer, MED12, CDK8, tethering luciferase assay, transcription regulation, directed affect, lncRNA

## Abstract

In recent years, long noncoding RNAs (lncRNAs) have received increasing attention and have been reported to be associated with various genetic abnormalities. However, the functions of many lncRNAs, including those of long exonic noncoding RNAs (lencRNAs), have not yet been elucidated. Here, we used a novel tethering luciferase assay to analyze the transcriptional regulatory functions of five lencRNAs that are upregulated in cancer. We found that the lencRNA *PRKDC-210* interacts with MED12, a component of the CDK8 complex, to regulate the transcription of several genes. The transcriptional activation ability of *PRKDC-210* was abolished in siRNA-treated CDK8-depleted cells. We also confirmed the enrichment of *PRKDC-210* on RNA polymerase II. RNA-seq analysis of cells in which *PRKDC-210* or *PRKDC* mRNA was knocked down using antisense oligonucleotides revealed that *PRKDC-210* can affect the expression levels of genes related to fatty acid metabolism. Finally, we used a ChIRP assay to examine *PRKDC-210*-enriched sites in the genome. Overall, our findings demonstrate that the lencRNA *PRKDC-210* promotes transcription through the CDK8 complex pathway at the transcription initiation site. We propose that *PRKDC-210* can affect the transcription of adjacent genes after its transcription and splicing.

## 1. Introduction

Noncoding RNAs (ncRNAs) are ubiquitous in eukaryotes, and those larger than 200 nt are classified as long noncoding RNAs (lncRNAs) [1,2]. According to their genomic position, lncRNAs are further classified into five types, namely, intronic, exonic, intergenic, antisense, and overlay, although the vast majority of lncRNA research has focused on long intergenic noncoding RNAs (lincRNAs) [3,4,5]. In recent years, lncRNAs have been reported to perform various functions in cells through RNA–protein interactions [6,7] and are also associated with genetic abnormalities in diseases such as cancer [8,9].

Long exonic noncoding RNAs (lencRNAs), which contain retained introns, have not received much attention to date. As they are splice variants of mRNAs, detection and recognition of lencRNAs are difficult; however, advancements in RNA detection technologies have revealed that lencRNAs are indeed retained in cells [10]. Consequently, we assumed that lencRNAs have specific cellular functions [11,12]. Recent studies have shown that several ncRNAs are associated with cancer [13,14]. In addition, a number of lncRNAs interact with transcriptional regulatory proteins to regulate gene transcription [6,7,11,15]. Therefore, we postulated that, after being spliced, lencRNAs may also affect the transcription of adjacent genes, resulting in abnormal gene expression in some genetic disorders.

Here, we designed a tethering luciferase assay system to detect the effects of lencRNAs on gene transcription. This system was used to examine the abilities of five selected lencRNAs (*BAP1-206*, *PRKDC-210*, *TP53-215*, *PIK3R1-206*, and *PIK3R1-209*), all of which are expressed at high levels in cancer cells [10]. Mediator complex subunit 12 (MED12) has been reported as a potential biomarker of cancer [16] and is a constituent of the CDK8 complex, which is involved in RNA pol II-mediated transcription. A past report demonstrated that MED12 interacts with *ncRNA-A7* to upregulate gene transcription [17].

The results of the tethering luciferase assay and RNA immunoprecipitation (RIP)-qPCR assays revealed that the lencRNA *PRKDC-210* interacts with MED12 to drive gene transcription, and this effect was abolished in cells lacking CDK8. The effects of endogenous *PRKDC-210* on gene transcription were also examined using RNA-knockdown, RNA-sequencing (RNA-seq), and chromatin isolation by RNA purification (ChIRP) assays.

## 2. Results

### 2.1. Tethering Luciferase Assay System

**Figure 1:**
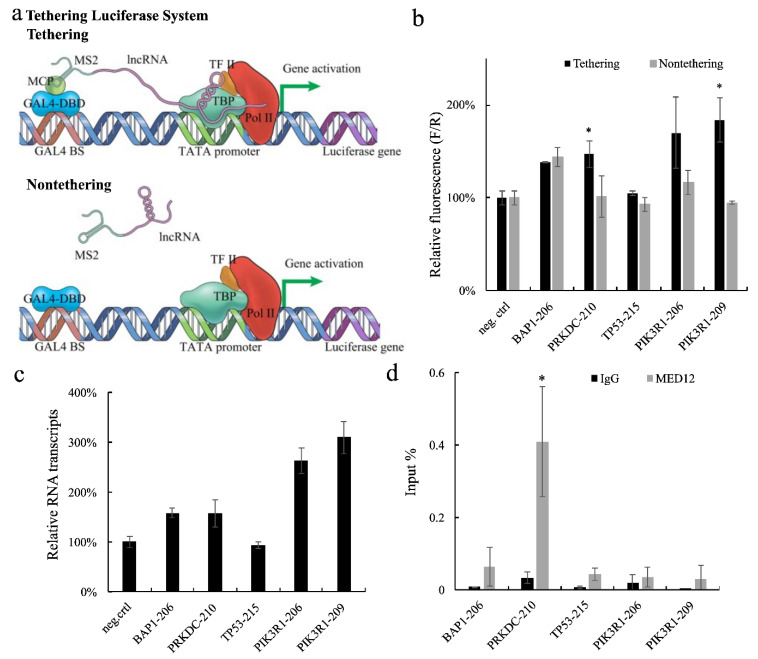
(**a**) Schematic illustration of the tethering luciferase assay system. (**b**,**c**) Tethering luciferase assay using HEK293T cells, relative fluorescence and mRNA levels were detected. We used the empty MS2 plasmid instead of the MS2 lencRNA plasmid as negative control (neg.ctrl). Value = sample/neg.ctrl. (**b**) We used the Dual-Luciferase^®^ Reporter assay to detect the results of the tethering assay; the luciferase gene expression levels of different lencRNAs in the tethering or nontethering were compared. F/R, Firefly luciferase/Renilla luciferase. (**c**) Total RNA was extracted from cells transfected with tethering luciferase assay plasmid, and the differences of luciferase mRNA transcript between different lencRNAs were compared. Total RNA was reverse-transcribed into cDNA using random primers and quantified by qPCR using specific primer for luciferase gene. (**d**) The binding of different lencRNAs to MED12 in MCF7 cells was compared by RIP-qPCR assay. Value = sample/input. Data in all panels are represented as the mean  ±  s.e.m. of three independent experiments. * *p*  <  0.05 by a two-tailed Student’s *t*-test (compared with nontethering (**a**) or IgG (**d**)).

To examine whether lencRNAs can affect gene transcription, we designed a novel screening technique called the tethering luciferase assay system (Figure 1a). This system is divided into three parts: a lencRNA bound to MS2 RNA (MS2-lencRNA), a fusion protein comprising the MS2 coat protein (MCP) and the GAL4 DNA binding domain (MCP-GAL4DBD), and a luciferase reporter gene containing a GAL binding site. The lencRNA is tethered upstream of the luciferase gene by the interaction between MS2 and MCP-GAL4DBD, to construct an enhancer-like structure or cofactor recruiting structure. A TATA box is used as the promoter for the reporter gene because only a few factors bind to this type of element [18,19], which makes it easier to evaluate the role of the lencRNA in transcriptional regulation. The GAL4DBD protein (without MCP), which was unable to tether the lencRNA upstream of the luciferase gene, was used as a nontethering control. Using this system, we anticipated that the tethered lencRNA would be enriched upstream of the luciferase gene, thereby enabling it to regulate transcription of the reporter gene. By contrast, in the nontethering control, the lencRNA would be expected to float in cells and have little effect on transcription of the luciferase gene. We used HEK293T cells for all tethering luciferase assays because HEK293T is highly transfected and easy to culture.

### 2.2. Identification of Long Exonic Non-Coding RNA with Transcriptional Regulatory Functions

We selected five genes with high intron retention in cancer and selected their lencRNA variant from the gene bank (http://asia.ensembl.org/index.html, accessed on 2 October 2019). The *BAP1-206*, *PRKDC-210*, *TP53-215*, *PIK3R1-206*, and *PIK3R1-209* lencRNAs were inserted into plasmids containing MS2 RNA sequences, and the empty MS2 plasmid was used as a negative control. Plasmids harboring the three components of the tethering luciferase assay system were then co-transfected into HEK293T cells, and relative luciferase activities were examined in cells expressing the tethered or nontethered lencRNAs. The relative luciferase activity in cells expressing tethered *PRKDC-210* or *PIK3R1-209* was significantly higher than that in cells expressing the nontethered versions of these lencRNAs (Figure 1b). Moreover, compared with that in negative control cells, the luciferase mRNA level was higher in cells expressing *BAP1-206*, *PRKDC-210*, *PIK3R1-206*, or *PIK3R1-209* (Figure 1c). The enhancement level of mRNA detection was generally higher than that of the luciferase assay. We guessed that the difference should be caused by different detection objects and methods, as well as uncontrollable factors in the process of transcription to translation. Moreover, the higher transcription levels confirmed what we suspected, namely, that the regulatory function of these lencRNAs occurs at the transcription level.

Next, we performed a RIP-qPCR assay of *human* breast cancer MCF7 cells using an anti-MED12 antibody, because these lencRNAs are present in relatively high levels in human breast cancer cells [10]. The results indicated that *PRKDC-210* can interact with MED12 or CDK8 complex (Figure 1d). Because the TATA promoter is not regulated by MED12 or its complexes, we hypothesized that *PRKDC-210* promotes the enrichment of MED12 or its complex, thereby enabling it to affect RNA pol II activity. Therefore, we focused our study on the lencRNA *PRKDC-210*.

### 2.3. The long exonic non-coding RNA PRKDC-10 Promotes Activity of the CDK8 Complex to Drive Transcription

**Figure 2:**
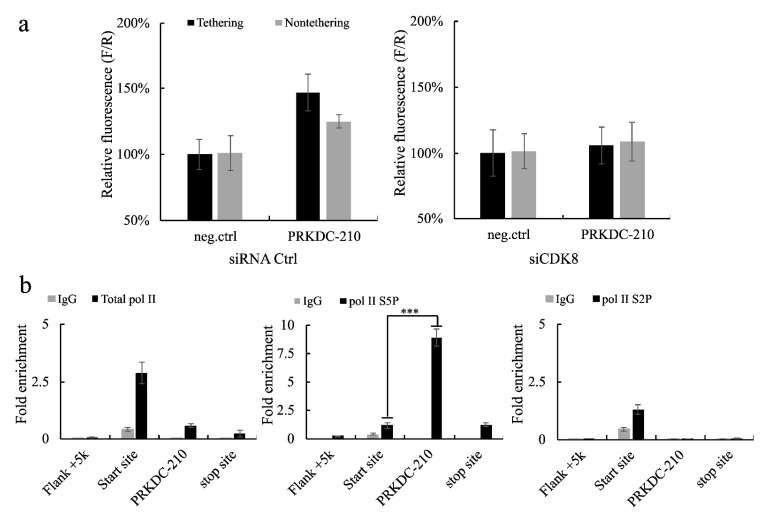
(**a**) This figure shows the expression of luciferase after tethering of *PRKDC-210* in control siRNA-treated HEK293T cells and siCDK8-treated HEK293T cells. F/R, firefly luciferase/Renilla luciferase. (**b**) RIP-qPCR assays of MCF7 cells using antibodies targeting total RNA pol II, RNA pol II S5P, and RNA pol II S2P. The enrichment of *PRKDC* mRNA at different sites and the enrichment of *PRKDC-210* in different phosphorylation of RNA Pol II are shown. Data in all panels are represented as the mean  ±  s.e.m. of three independent experiments. *** *p*  <  0.001 by a two-tailed Student’s *t*-test.

As a constituent of the CDK8 complex, MED12 plays a role in activating phosphorylation of RNA pol II at Ser 5 to promote transcription [20]. Given that the RIP-qPCR analysis identified an interaction between *PRKDC-210* and MED12, we hypothesized that *PRKDC-210* may also affect the activity of the CDK8 complex. To test this hypothesis, we knocked down intracellular CDK8 expression in HEK293T using a predesigned siRNA and performed a tethering luciferase assay in CDK8-depleted HEK293T cells. The ability of *PRKDC-210* to upregulate luciferase activity was abolished in CDK8 depleted cells (Figure 2a).

Next, we performed RIP-qPCR assays of MCF7 cells using antibodies targeting total RNA pol II and RNA pol II phosphorylated at Ser 5 (S5P) or Ser 2 (S2P). After immunoprecipitation, RT-qPCR was used to analyze various regions of the gene encoding *PRKDC*, including the 5 kbp upstream flanking region (used as a negative control), the transcription start site (TSS; used as a positive control), *PRKDC-210*, and the stop site. The results indicated that *PRKDC-210* was enriched on RNA pol II S5P (Figure 2b). At the initiation of transcription, the CDK8 complex activates the phosphorylation of RNA pol II at Ser 5 [20]; therefore, taken together with our previous findings, these results suggest that *PRKDC-210* may act as a linker between the MED12–CDK8 complex and/or the CDK8–RNA pol II S5P complex to promote transcription.

### 2.4. Functions of PRKDC-210 in MCF7 Cells

**Figure 3:**
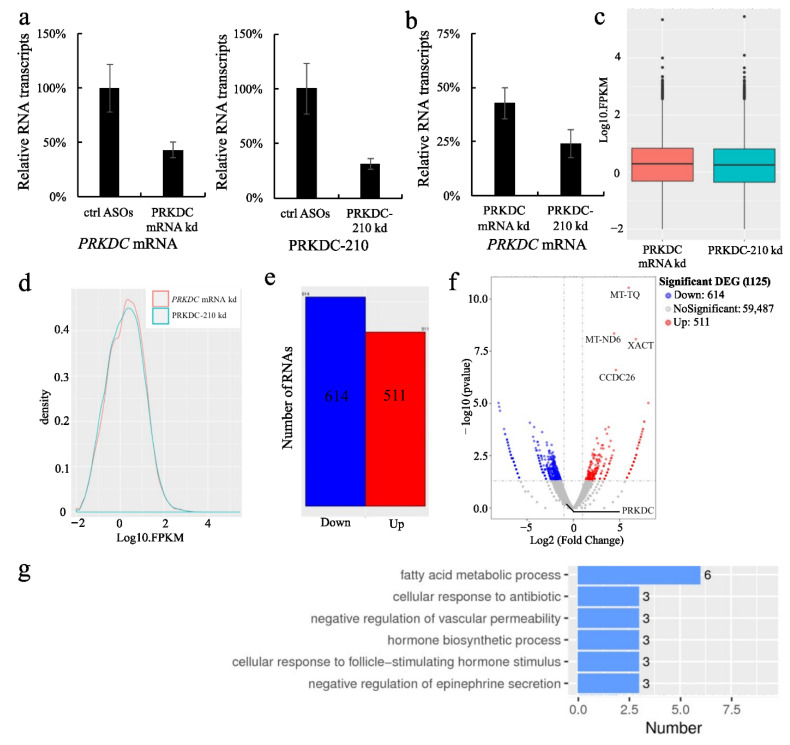
(**a**,**b**) Total RNA was extracted from MCF7 cells expressing control (ctrl) ASOs or ASOs targeting the *PRKDC* mRNA or *PRKDC-210*, and the RNA transcripts levels were detected via RT-qPCR. Kd means knockdown. Data are represented as the mean  ±  s.e.m. of three independent experiments. (**c**–**g**) RNA-seq analyses of *PRKDC* mRNA knockdown and *PRKDC-210* knockdown cells. (**c**,**d**) FPKM profiles showing the overall gene expression levels. (**e**) The numbers of RNAs with altered transcript levels in *PRKDC-210* knockdown cells versus control cells. (**f**) Genes that were significantly differentially expressed (fold change >2 and *p* < 0.05) in *PRKDC-210* knockdown cells versus *PRKDC* mRNA knockdown cells. (**g**) Gene ontology biological process enrichment analysis.

To further analyze its cellular function, we used an antisense oligo (ASO) to knock down endogenous *PRKDC-210* in MCF7 cells. In addition, *PRKDC* mRNA expression was also knocked down. RT-qPCR analyses performed 24 h after transfection with each ASO confirmed successful knockdown of both *PRKDC-210* and the *PRKDC* mRNA (Figure 3a). Notably, the expression level of the *PRKDC* mRNA was lower in cells expressing the *PRKDC-210* ASO than in those expressing the *PRKDC* mRNA ASO (Figure 3b).

Next, we performed RNA-seq analysis of total RNA extracts from *PRKDC-210* knockdown and *PRKDC* mRNA knockdown cells. This analysis confirmed the reduction in *PRKDC* mRNA expression following knockdown of *PRKDC-210* (Table S1). There was no significant difference between overall RNA transcription in *PRKDC-210* knockdown and *PRKDC* mRNA knockdown cells, and only a few RNAs were affected by *PRKDC-210* knockdown (Figure 3c,d). Compared with those in *PRKDC* mRNA knockdown cells, the expression levels of 614 and 511 RNAs were downregulated and upregulated, respectively, by knockdown of *PRKDC-210* (Figure 3e,f). A gene ontology (GO) analysis of the affected RNAs indicated that *PRKDC-210* may affect the expression of fatty acid metabolism-related genes (Figure 3g). Notably, fatty acid metabolism is an additional energy supply pathway in cancer cells [21].

### 2.5. PRKDC-210 Acts on the Endogenous Genome

**Figure 4:**
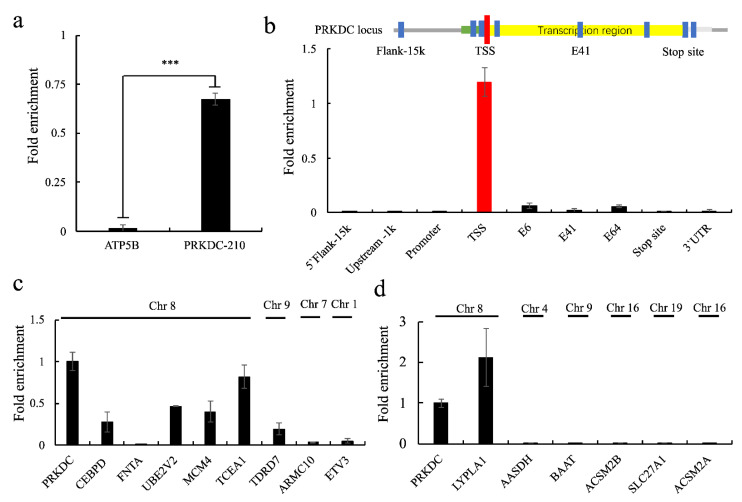
MFC7 was used for all ChIRP assays. (**a**) RT-qPCR analyses of *PRKDC-210* and ATP5B (negative control) levels in the RNA precipitated by ChIRP. (**b**–**d**) The DNAs associated with the immunoprecipitated RNAs in the ChIRP assay were analyzed by qPCR using specific primers. (**b**) The distribution of *PRKDC-210* at the *PRKDC* locus. (**c**) Enrichment of *PRKDC-210* at the TSSs of various genes. (**d**) Enrichment of *PRKDC-210* at the TSSs of genes related to fatty acid metabolism. Data in all panels are represented as the mean  ±  s.e.m. of three independent experiments. *** *p*  <  0.001 by a two-tailed Student’s *t*-test.

Given its interaction with the CKD8 complex and RNA pol II S5P, we hypothesized that *PRKDC-210* would be enriched at the promoter or transcription initiation regions of genes. To examine this possibility, we used a ChIRP assay to assess RNA–DNA binding [22]. RT-qPCR analysis confirmed that *PRKDC-210* was precipitated by the ChIRP probe set (Figure 4a). We suspected that *PRKDC-210* would act on adjacent genes, so we examined enrichment of various regions of the *PRKDC* locus on the immunoprecipitated *PRKDC-210* RNA. We examined the 15 kbp upstream flanking region (used as a negative control), 1 kbp upstream site, promoter region, transcription start site (TSS), Exon region (6, 41 and 64), transcription stop site and the 3’ UTR, respectively. As expected, *PRKDC-210* was enriched at the TSS of the *PRKDC* gene (Figure 4b).

Next, to determine whether *PRKDC-210* can act directly on genes other than *PRKDC*, we performed ChIRP-qPCR analyses of eight randomly selected genes that were detected as differentially expressed in the RNA-seq analyses following knockdown of *PRKDC-210*. Among them, *CEBPD*, *FANTA*, *UBE2V2*, *MCM4* and *TCEA1* are located on the same chromosome as *PRKDC* (chromosome 8), whereas *TDRD7*, *ARMC10*, and *ETV3* are located on chromosomes 9, 7 and 1, respectively. *PRKDC-210* was enriched at the TSSs of the *CEBPD*, *UBE2V2*, *MCM4*, *TCEA1* and *TDRD7* genes (Figure 4c). We also performed a ChIRP-qPCR analysis of the TSSs of genes included in the GO fatty acid metabolic process, including *LYPLA1* (chromosome 8), *AASDH* (chromosome 4), *BAAT* (chromosome 9), *ACSM2B* (chromosome 16), *SLC27A1* (chromosome 19) and *ACSM2A* (chromosome 16). *PRKDC-210* was enriched at the *LYPLA1* gene only (Figure 4d). Overall, these results suggest that genes on chromosome 8 are more likely to be targeted by *PRKDC-210*.

## 3. Discussion

In the past, limitations in detection technologies have meant that the roles of lencRNAs have been overlooked. In particular, as lencRNAs are splice variants of mRNAs, they are often confused with mRNAs during analyses. Here, we designed a novel tethering luciferase assay to detect the regulatory functions of exogenous lencRNAs in transcription and found that the lencRNA *PRKDC-210* interacts with MED-12 and/or the CDK8 complex to upregulate gene expression. Since the tethering luciferase assay utilizes a TATA box, which is not associated with MED12 enrichment, we assumed that *PRKDC-210* promoted the interaction of MED12 and its complex with RNA polymerase II S5P to drive transcriptional upregulation. In addition to *PRKDC-210*, the lencRNAs *BAP1-206*, *PIK3R1-206* and *PIK3R1-209* also increased transcription of the luciferase gene in our initial screen (Figure 1b,c). We did not analyze the functions of these lencRNAs further because they were unable to interact with MED12 (Figure 1d); however, their potential regulatory roles and interaction with other transcriptional complexes will be examined in the future.

An interesting phenomenon was observed in the ChIRP assay. Most of the genes affected by *PRKDC-210* are located on chromosome 8, which is where the *PRKDC* locus is situated (Figure 4c,d). Among the 14 genes examined in the ChIRP assay (eight randomly selected genes and six fatty acid metabolic process-related genes), *CEBPD*, *FNTA*, *UBE2V2*, *MCM4*, and *LYPLA1* are located on chromosome 8, which has a total length of 146 Mbp. The maximum distance between these genes and the *PRKDC* locus is about 6.5 Mbp. Notably, the *CEBPD* and *MCM4* loci are located <50 Kbp and 7 bp from the *PRKDC* locus, respectively. The pairwise genetic distances were calculated according to Rutgers Map v.3, a combined linkage-physical map of the human genome (http://compgen.rutgers.edu/map_interpolator.shtml, accessed on 27 September 2022). The minimal genetic distances of intergenic recombinations were obtained for *PRKDC*, *CEBPD*, *MCM4*, and *UBE2V2* (<0.05 cM). The genetic distance between *FNTA* and *PRKDC* was about 0.5 cM. In general, linkage disequilibrium is supposedly noticeable at such short distances. *PRKDC* is located far from *LYPLA1*, with a genetic distance of about 5.0 cM. Unlike *CEBPD*, *MCM4*, and *UBE2V2*, *LYPLA1* does not appear to display linkage disequilibrium with *PRKDC*. Nonetheless, *LYPLA1* was also affected by *PRKDC-210*. In addition, because chromosomes move randomly in the nucleus, the relative positions of two genes on different chromosomes are uncertain, while the relative positions of two genes on the same chromosome do not change significantly. Of course, this could be due to chromosome folding or another configuration that causes *PRKDC-210* to enhance transcription within a certain range of chromatin 8 [23]. However, heterochromatin structure should not be associated with the transcriptional enhancement of *PRKDC-210*, as heterochromatin structure is thought to be unable to express genes [24]. We propose that genes on the same chromosome as the *PRKDC* locus may be more susceptible to *PRKDC-210* than those on other chromosomes, as after transcription and splicing, *PRKDC-210* may be bound rapidly by nearby CDK8 complexes or RNA pol II, which then act on neighboring genes.

Based on the results of the tethering luciferase assay and RIP-qPCR analyses (Figure 1 and Figure 2), we speculate that *PRKDC-210* acts directly on the CDK8 complex in the nucleus. Notably, lencRNAs such as *PRKDC-210* are not modified by poly(A) tailing; hence, they may act rapidly on nearby proteins and promote gene expression immediately after their generation. The regulatory action of lencRNAs may be a cause of abnormal gene expression in cancer. The lencRNAs examined in our current study are all expressed at high levels in cancer. As a product of alternative splicing, the increased generation of lencRNAs is likely attributable to genetic abnormalities. Indeed, there have been numerous reports of cancers producing abnormal mRNA splicing [10,25,26]. Although these reports have focused mainly on protein-coding RNAs, there are also reports confirming that lncRNAs are spliced from coding genes [27]. More in-depth studies are required to analyze the mechanisms underlying the production and functions of lencRNAs. Such studies may expand our current understanding of diseases caused by splicing abnormalities and lay the foundation for future therapeutic developments.

In conclusion, lencRNA is a type of lncRNA produced by abnormal splicing caused by genetic deviations such as cancer. Here, we found that the lencRNA *PRKDC-210* interacts with the CDK8 complex, resulting in transcriptional upregulation, which may be a major cause of abnormal gene expression in cancer. Additional studies are needed to understand the roles of lencRNAs in disease states.

## 4. Materials and Methods

### 4.1. Cell Lines

Two cell lines, HEK293T and MCF-7, were used in this study. HEK293T was used for all tethering luciferase assays for detecting exogenous lencRNA. MCF-7 cells were used to detect endogenous lencRNA. It was used for knockdown assay, RNA immunoprecipitation assay, RNA-Seq and chromatin isolation by RNA purification assay, respectively.

### 4.2. Cell Culture

HEK293T cells and MCF-7 cells were cultured in high-glucose DMEM (Nacalai Tesque, Kyoto, Japan) supplemented with 10% FBS (BioWest, Funakoshi, Japan). The cells were maintained in a 5% CO_2_ incubator at 37 °C [28].

### 4.3. Transfection

Twenty-four hours before transfection, cells were seeded into 12-well cell culture plates (TrueLine, New York, NY, USA) at a density of 2–3 × 10^5^ cells/well. The culture medium was replaced with 1 mL of fresh medium before transfection.

For the tethering luciferase assay in HEK293T cells, a mixture containing 400 ng of the Firefly luc plasmid, 10 ng of the Renilla luc plasmid, 50 ng of the MCP-GAL4 plasmid, 50 ng of the MS2-lencRNA plasmid, 2.5 µL of polyethylenimine (1.0 µg/µL; Polysciences, Warrington, PA, USA), and 100 µL of OPTI-MEM (Thermo Fisher Scientific, Waltham, MA, USA) was incubated at room temperature for 20 min. Subsequently, the transfection master mix was added to the cell culture medium, and cells were incubated for 24 h [29,30].

For siRNA transfections, HEK293T cells were seeded into 12-well plates at a density of 1 × 10^5^ cells/well. Following the manufacturer’s protocol, 2 µL of Lipofectamine^TM^ RNAiMAX (Thermo Fisher Scientific) and 24 pmol of siRNA were added to 100 µL of OPTI-MEM, and the mixture was incubated at room temperature for 20 min. The transfection master mix was then added to the cell culture medium, and the tethering luciferase assay was performed 24 h later [31]. The Mission^TM^ CDK8 siRNA (SASI_WI_00000016) and control siRNA (Universal Negative Control #1) were obtained from Sigma Aldrich (Burlington, MA, USA).

For knockdown of *PRKDC* mRNA and *PRKDC-210* in MCF-7 cells, following the manufacturer’s protocol, 5 µL of Lipofectamine^TM^ 2000 (Thermo Fisher Scientific) was mixed with 100 pmol of ASOs in 100 µL of OPTI-MEM and incubated at room temperature for 5 min. The transfection master mix was then added to the cell culture medium, and the cells were incubated for 24 h [32]. Phosphorothioate-modified ASOs were obtained from Eurofins Genomics (Tokyo, Japan).

### 4.4. Dual-Luciferase Reporter Assay

The Dual-Luciferase^®^ Reporter Assay System (Promega, Madison, WI, USA) was used to detect luciferase activity in the tethering luciferase assay. After transfection for 24 h, the culture medium was removed, and the cells were washed once with PBS(-) (Nacalai Tesque). Subsequently, 250 µL of 1× passive lysis buffer was added, and the cells were shaken for 15 min at room temperature. To detect the firefly luciferase signal, a 10 µL aliquot of the lysed cells was added to a luminometer tube along with Luciferase Assay Reagent II. Subsequently, Stop & Glo^®^ Reagent was added to the tube to enable detection of the Renilla luciferase signal. The ratio of firefly to Renilla luciferase was then determined [33]. The signals were detected using a Gene Light 55 luminometer (WAKENYAKU, Kyoto, Japan) with the following detection conditions: delay time 3 s, count time 6 s, repeat time 1.

### 4.5. RNA Extraction

RNA extraction was performed using TRIzol^®^ reagent (Thermo Fisher Scientific), according to the manufacturer’s instructions. Briefly, the culture medium was removed, and the cells were washed once with PBS(-), followed by addition of 400 µL of TRIzol^®^ reagent and RNA extraction using the basal protocol [34].

### 4.6. RT-qPCR

Total RNA (500 ng) was reverse transcribed into cDNA using ReverTra Ace^®^ reagent (TOYOBO, Osaka, Japan) and random primers (5′-NNNNNNNNN-3′). In the RIP assays, the RNA pellet was dissolved in nuclease-free water, and the entire sample was used for reverse transcription.

The qPCR assays were performed using TB Green^®^ Premix Ex Taq™ II (Tli RNaseH Plus) (TaKaRa, Kusatsu, Japan), according to the manufacturer’s protocol, and primers were obtained from Eurofins Genomics. Analyses were performed using an Mx3000p^TM^ qPCR system (Agilent, Santa Clara, CA, USA) [35].

### 4.7. RNA Immunoprecipitation

Cells were grown to 80–90% confluency in 10 cm culture dishes. The medium was then changed, and the cells were incubated for 30 min at 37 °C. Subsequently, the medium was removed, and the cells were washed three times with PBS(-); then, 5 mL of 1 × nuclear isolation buffer (3 mL of Milli-Q water, 1 mL of PBS, 1 mL of 5 × NIB (1.28 M sucrose, 40 mM Tris-HCl pH 7.5, 20 mM MgCl_2_, 4% Triton X-100)) was added. The cells were then scraped from the dish, transferred to a 15 mL centrifuge tube, placed on ice for 20 min, and centrifuged for 15 min at 2000 RCF. Next, the supernatant was removed, and the pellet was resuspended in 500 µL of RNA isolation buffer (150 mM KCl, 25 mM Tris-HCl pH 7.5, 5 mM EDTA, 0.5% NP-40, 0.5 mM DTT, protease inhibitor cocktail, RNase inhibitor, DNase I) using a 25 G syringe tip. After centrifuging at 12,500 RCF for 10 min, the supernatant was collected, and a 50 µL sample (used as input) was stored at 4°C. Another 400 µL sample was mixed with 2 µg of the specific antibody (Abcam, Cambridge, UK) and 10 µL of Dynabeads™ Protein G (Thermo Fisher Scientific), and then incubated overnight at 4°C with gentle rotation. The next morning, the supernatant was separated from the beads using a magnetic device, and the pelleted beads were washed three times with RNA isolation buffer and once with PBS(-) [36]. Finally, 400 µL of TRIzol^®^ reagent was added to the input sample and beads, and RNA extraction was performed as described in Section 4.4.

### 4.8. RNA Sequencing

Total RNA was extracted from cells transfected with ASOs as described in Section 4.4. Subsequently, the samples were analyzed using the RNA-seq service of Azenta Life Sciences (Tokyo, Japan). The level of gene expression was indicated by the read density and calculated as shown below (where FPKM represents the fragments per kilo bases per million reads) based on read counts from HT-seq (V. 0.6.1) [37].

$$\mathrm{FPKM}=\frac{\mathrm{total}\;\mathrm{exon}\;\mathrm{fragments}}{\mathrm{mapped}\;\mathrm{reads}\left(\mathrm{millions}\right)\times \;\mathrm{exon}\;\mathrm{length}\;\left(\mathrm{Kbp}\right)}$$

The ratio of total exon fragments to mapped reads is normalized to the gene length (exon length), such that the expression levels of genes with different sequencing depths and lengths are comparable. RNAs with reads > 10 and FPKM > 2 were screened as valid samples.

### 4.9. Chromatin Isolation by RNA Purification

The experimental method followed the protocol of Ci Chu et al. [22]. To isolate *PRKDC-210*, a probe set targeting *PRKDC-210* needed to be designed. Probes were designed on the *PRKDC-210* at about 100 bp intervals, with a total of 6 probes. The ChIRP-probe set was modified with 3’ BiotinTEG and ordered from Eurofins Genomics.

Cells were grown to 80–90% confluency in 10 cm cell culture dishes. The medium was then removed, and the cells were washed three times with PBS(-) before being resuspended in PBS(-), transferred to a 50 mL tube, and centrifuged. Next, the supernatant was removed, and the pellet was resuspended in 10 mL of 1% glutaraldehyde (prepared immediately before use) and incubated for 10 min at room temperature, with gentle shaking to crosslink the cells. To stop the crosslinking, 1 mL of 1.25 M glycine was added, and the sample was incubated for 5 min at room temperature. Subsequently, the tube was centrifuged, the supernatant was removed, the pellet was washed once with PBS(-), and the sample was then centrifuged again. Next, the pellet in PBS(-) was transferred to a 1.5 mL centrifuge tube, the PBS(-) was removed, and 500 µL of cell lysis buffer (50 mM Tris-HCl pH 7.0, 10 mM EDTA, 1% SDS, protease inhibitor cocktail, RNase inhibitor) was added. The sample was then sonicated until it became clear. A 10 µL aliquot of the lysate was reserved as the DNA/RNA input sample, and then 1 mL of hybridization buffer (750 mM NaCl, 1% SDS, 50 mM Tris-HCl pH 7.0, 1 mM EDTA, 15% formamide, protease inhibitor cocktail, RNase inhibitor) and 100 pmol of ChIRP probes (Eurofins Genomics) were added to the remaining sample, followed by incubation for 4 h at 37 °C with rotation. Next, Dynabeads Biotin Binder beads (100 µL; Thermo Fisher Scientific) were washed with cell lysis buffer, added to the sample, and incubated for 30 min at 37 °C with shaking. Subsequently, the beads were washed five times with 1 mL of wash buffer (2× SSC, 0.5% SDS) and then resuspended in 1 mL of wash buffer. A 100 µL aliquot was reserved for RNA isolation. The remaining 900 µL of the bead suspension was removed from the wash buffer and added to 300 µL of elution buffer to elute the DNA from the beads.

For the RNA component, the wash buffer was removed from the beads for treatment with proteinase K. After the addition of TRIzol^®^ reagent (as described in Section 4.4), RT-qPCR analyses were performed using specific primers for RNA products.

For the DNA component, the sample was treated with proteinase K. Subsequently, 300 µL of PCI (phenol: chloroform: isoamyl alcohol; 25:24:1) was added, the sample was shaken vigorously for 10 min, and then the supernatant was removed following centrifugation. Next, 30 µL of NaOAc and 900 µL of 100% EtOH were added, and the sample was incubated overnight at −20°C. The next morning, the supernatant was removed by centrifugation, and the DNA pellet was washed with 70% EtOH, centrifuged again to remove the supernatant, air-dried, and dissolved in Milli-Q water. qPCR analyses were performed using specific primers.

### 4.10. Primer

qPCR primers, Antisense Oligo DNA and ChIRP probes were all ordered from Eurofins Genomics; sequences can be found in Supplementary Data (Table S2).

## Data Availability

The data presented in this study are available on request from the corresponding author. The data are not publicly available due to the data will be used in subsequent studies.

## Supplementary Materials

The following supporting information can be downloaded at: https://www.mdpi.com/article/10.3390/ijms232213783/s1.
